# Nationwide audit of cervical cancer screening reveals unsatisfactory triage strategies and trends over time in incidence among cytology negative women

**DOI:** 10.1002/ijc.70415

**Published:** 2026-03-09

**Authors:** Cecilia Kärrberg, Penelope Gray, Kristina Elfgren, Hanna Milerad, Bengt Andrae, Jiayao Lei, Pär Sparén, Joakim Dillner, Jiangrong Wang, K. Miriam Elfström

**Affiliations:** ^1^ Department of Obstetrics and Gynecology, Institute of Clinical Sciences, Sahlgrenska Academy University of Gothenburg Gothenburg Sweden; ^2^ Department of Obstetrics and Gynecology Sahlgrenska University Hospital Gothenburg Sweden; ^3^ Center for Cervical Cancer Elimination, Department of Clinical Science Intervention and Technology, Karolinska Institutet Stockholm Sweden; ^4^ Regional Cancer Center Stockholm Gotland Sweden; ^5^ Division of Obstetrics and Gynaecology, Department of Clinical Science Intervention and Technology, Karolinska Institutet Sweden; ^6^ Department of Medical Epidemiology and Biostatistics Karolinska Institutet Stockholm Sweden; ^7^ Center for Cervical Cancer Elimination, Molecular Cancer Diagnostics & Screening Karolinska University Hospital Stockholm Sweden

**Keywords:** cervical cancer, HPV‐based screening, quality assurance, screening audit

## Abstract

Regular audits are essential for monitoring and evaluation of cervical screening. We audited the Swedish cervical screening program during the transition to HPV‐based screening using a registry‐based cohort design. In total, 5689 consecutive, confirmed cases of invasive cervical cancer between 2012 and 2022 were retrieved from the Swedish Quality Register for Gynecological Cancer. Their screening history, screening status, invitations to cervical screening, all diagnoses in screening cytology and HPV genotypes were retrieved from the Swedish National Cervical Screening Registry. Furthermore, all cancer cases during 2018–2022 were classified according to their screening history and the age‐standardized cervical cancer incidence rate stratified by screening history and calendar year was calculated among the cohort of all women in Sweden ages 29–84. Non‐participation remains the main process related history with high risk of cancer (cervical cancer incidence rate, IR, among non‐attenders was 33.9 [95% confidence intervals, 95% CI, 28.2–39.5] per 100,000 person‐years, /100,000PY, representing 31% of cases in 2022). The cervical cancer IR after negative cytology increased with calendar time (IR = 5.5 [95% CI, 4.1–6.8]/100,000PY in 2012 and 11.5 [8.1–14.9]/100,000PY in 2022) and was high after a positive HPV test followed by negative cytology triage (IR = 56.7 [39.9–73.6] in 2022). In conclusion, this comprehensive audit has identified a decreasing protection by the screening test under discontinuation (cytology) and that the performance as a triage test after HPV‐testing is unsatisfactory.

## Graphical abstract

What's new?

This population‐based cohort study aimed to demonstrate how annual audits of all aspects of the cervical cancer screening pathway can be performed using register national data with clinical validation to identify patterns of risk and inform program change. The audit of the Swedish cervical screening program during the transition from primary cytology‐ to human papilloma virus (HPV)‐based screening shows increasing incidence rates among cytology‐negative women, with a particularly high incidence among HPV‐positive women with negative cytology triaging. The findings reflect differing risks as screening methods change in a real‐life program and demonstrate aspects of the screening pathway that need optimization.

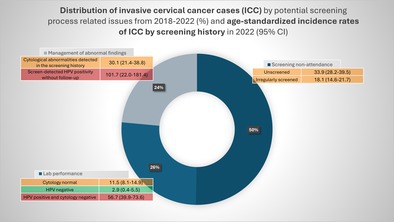

**Abbreviations**
AC: Adenocarcinoma
ASC: Adeno‐squamous carcinoma
EU: European Union
FIGO: International Federation of Gynecology and Obstetrics
HPV: Human Papilloma Virus
ICD: International Classification of Disease
IR: Incidence rate
NKCx: Swedish National Cervical Screening Registry
P: *p*‐value
SCC: Squamous cell carcinoma
SQRGC: Swedish Quality Registry for Gynecological Cancer
WHO: World Health Organization

## 1 INTRODUCTION

Since 1993, the European Union, EU, has released guidelines for quality assurance in cervical screening.^1^ These have been updated subsequently to reflect changes in cervical screening technologies and prevention strategies. Recurrent audits of the screening process including further management and treatment of abnormal findings have been part of the recommended quality assurance strategy in the European Guidelines for Quality Assurance in Cervical Cancer Prevention since 2008 and have been further emphasized in recent best practices documents from the International Agency for Research on Cancer.^2^, ^3^ Audits facilitate systematic monitoring and evaluation of cervical screening, laboratory performance, and clinical management with the goal of identifying areas for program improvement.

European Union (EU) recommendations apply to all member states, but actual implementation varies, thus routine auditing frameworks are not operational in all countries.^4^ In line with European recommendations, the National Standard of Care in Sweden recommends annual routine program and laboratory audits of all cervical cancer cases.^5^ We have performed population‐based audits in the research setting to examine screening history and status of cervical cancer cases.^6^ In these analyses, we have used comprehensive and nation‐wide quality registers that contain complete data on cancer cases and screening histories to evaluate and quantify process related issues; the effectiveness of screening regarding participation, laboratory performance and clinical management of positive findings, in relation to age, histological type of cancer, and FIGO stage as well as the mode of detection.^7^ To provide annual feedback to the screening program, a protocol for routine annual audits of cervical cancer cases, using the same data sources for cancer cases and general population, was established to achieve evidence for improvements. This protocol involves key actors in the screening and clinical management, that is, the screening organization, the laboratories, gynecologists performing management of abnormalities, and directly links back to provide evidence for program development in the National Standard of Care.

Cervical screening programs should move from primary cytology‐based to primary HPV‐based screening. To capture the impact over time with increasingly sensitive primary screening methods, monitoring the process effectiveness at the population level is necessary. This enables the identification of groups of persistent risk allowing for the informed calibration of the program to reach cervical cancer elimination targets.^8^ Rather than utilizing a case–control design (reporting relative risks) when conducting the cervical screening audit as is commonly performed, we propose to switch to a cohort study design (reporting incidence rates as absolute risks by screening history and status), because given the constant change of screening modality, there is no longer a reference group with a stable risk profile over time. This population‐based cohort study aims to demonstrate how annual, nationally coordinated audits of all aspects of the screening chain can be performed using register data with clinical validation to identify patterns of risk and inform program change.

## 2 MATERIALS AND METHODS

### 2.1 Study setting

In Sweden, organized screening was initiated in the late 1960s. The program is regulated by national recommendations for screening modality, age, and intervals and implemented independently by regions that are responsible for invitations, testing, and follow‐up management. Coverage has remained relatively stable in recent decades with close to 80% of screening‐eligible women participating within the recommended interval.^9^

Following evidence on the superiority of HPV‐based screening compared to cytology from the European trials,^10^ the program guidelines were updated in 2015 to reflect a switch to HPV‐based screening for women ages 30–70 years old. Implementation of HPV‐based screening was gradual over 6 years with the last region switching in 2021. Before 2015, screening intervals were 3 years and 5 years for women below age 50 and women above age 50, respectively. In 2015, the interval was lengthened to 7 years for women ages 50–70 once screened HPV‐negative but remained 3 years for women ages 23–50 (this reflected cytology for women 23–29 and HPV for women over 30). These guidelines have since been updated again in 2022.

### 2.2 Cervical cancer cases and classification of screening status and history

**FIGURE 1:**
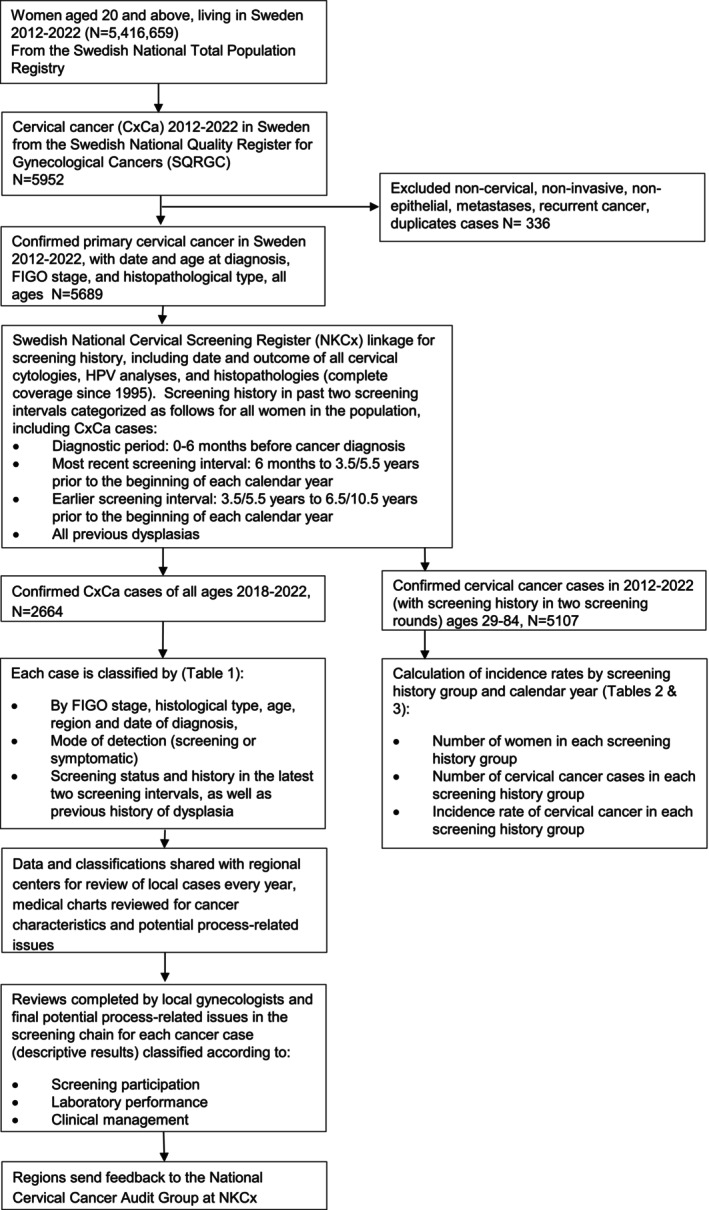
Study flowchart including data linkages and analyses.

Cases of invasive cervical cancer (ICD‐10 C53) were obtained from the Swedish Quality Registry for Gynecological Cancer (SQRGC), a register based on mandatory notification of cervical cancer cases when diagnosed by clinicians and pathologists. Only primary cervical cancers were registered in the audit. Exclusion criteria were high grade dysplasia, metastases in the cervix, non‐epithelial tumors, non‐cervical tumors, and recurrences of cervical cancer. For the analysis, the FIGO‐stage was classified into three groups: IA, IB and II+.^6^, ^11^, ^12^ The histopathological type of cervical cancer was categorized into four groups: squamous cell carcinoma (SCC), adenocarcinoma (AC), adeno‐squamous carcinoma (ASC), and rare histopathological types (e.g. neuroendocrine tumors, small cell carcinomas) (Figure 1).^11^

The classification of screening status and history was completed by individually linking all confirmed cervical cancer cases with their screening history in the Swedish National Cervical Screening Registry (NKCx). The NKCx contains all invitations to screening, all cervical screening results (cytology and HPV from screening, clinician taken samples as well as self‐samples), and all results from cervical histopathologies (including biopsies and excisional treatments).^13^ Screening status (screening invitations and participation) and screening history (screening test results) were evaluated for the two preceding intervals prior to cancer diagnosis. We have previously shown that screening participation and results in the two intervals preceding a cancer diagnosis are meaningful to examine with regard to associated risk.^7^, ^14^ Screening history according to age was estimated as having a screening test in the two recommended intervals prior to cancer diagnosis and for women above the screening target age range, having a screening test in the last two intervals before exiting the program. All samples taken, both organized and non‐organized, are captured in the screening history calculations. A sample taken within 6 months before diagnosis of cervical cancer was not classified as a screening sample.^7^ By reviewing the screening history in the two screening intervals of each cervical cancer case, we classified each case according to three main potential screening process‐related issues to descriptively highlight the distribution of cases by aspects of the screening chain that could be further optimized. The categories were as follows: screening participation, laboratory performance, clinical management.

Once the screening status and history was determined for each cancer case, further details on cases were collected as follows. Cases diagnosed in 2018–2022 underwent an initial review by the authors. Identified cases including case information (age at diagnosis, FIGO‐stage, histopathological type), screening status and history were then reviewed annually by experienced gynecologists responsible for the local management of dysplasia. These gynecologists reviewed the cases according to a standardized protocol by comparing the register‐based data to clinical medical charts, and the number of cases reviewed by each varied depending on the population size of the region. Parameters such as detection mode, diagnosis date (date of the first biopsy showing invasive cervical cancer), differentiation grade, and treatment modality of cervical cancer derived from individual linkage to the Swedish National Cervical Screening Registry (NKCx, more on this below) were checked against the medical charts. In all cases, reflective comments were added to help classify the screening history and status into potential screening process related issues (descriptive analysis outlined below). Collection of further details on the clinical work‐up of the cases and whether guidelines had been followed was facilitated through this chart review. Additional, nuanced information such as whether the case was pregnancy‐related or a stump‐cancer could be added through the medical chart review (since this is not available in the registers). The re‐review of cases in 2018–2022 showed that data recorded in SQRGC is mostly representative of the medical charts in terms of case definition, date of diagnosis, histopathological classification, and FIGO stage (data not shown) (Figure 1).

To explore the cervical cancer incidence rate in the population by screening history and status, cervical cancer cases in 2012–2017 were also included in the cohort analysis. These cases did not undergo medical chart re‐review as did cases from 2018 to 2022 but were linked to the NKCx for screening history and status. Using the three categories listed above, we further specified screening history into detailed categories to examine cancer incidence over time and within group (Table 1). Screening intervals were defined as 3 years for women under 50 and 5 years for women 50 and over according to recommendations at the time. The groups were defined to be mutually exclusive, so that for example, women who missed one screening round but had a positive test result in the next round were classified to test positive groups instead of non‐attending groups (Table 1). The screening status and history for all women living in Sweden in each year from the 1st of January 2012 to the 31st of December 2022 was determined using an extract from the Swedish Total Population Register and linkages to the NKCx. By doing so, incidence rates per group could be calculated at the population level, as described below.

**TABLE 1:** Classification of screening status and history.

Classification	Description
Unscreened	Women who did not have a screening test on record in the past two intervals
2 Irregularly screened	Women who did not attend screening in both past two intervals or had only a cytology normal test result in one interval
3 Cytology normal	Women who attended in both two intervals and had only cytology normal results (no HPV test, and no cytology abnormality)
4 HPV negative in both intervals	Women who attended in both two intervals and had negative HPV results in each interval (no HPV positive nor cytology abnormality)
5 HPV negative in one interval	Women who had ever had an HPV negative test result in past two intervals (no HPV positive nor cytology abnormality)
6 HPV positive and cytology negative	Women who had HPV positive result with only cytology normal results in past two intervals (no cytology abnormality)
7 Screen‐detected abnormality	Women who had any cytology abnormal result(s) in past two intervals, including abnormalities from cytology‐primary screening and the cytology triage in HPV‐based screening
8 Screen‐detected HPV positivity without follow‐up	Women who had HPV positive result but no cytology or histopathology test nor HPV negative result in past two intervals

### 2.3 Statistical analysis

We calculated the absolute number and the proportion of women aged 29 to 84‐years‐old residing in Sweden belonging to each screening history category and the absolute number cervical cancer cases per group by year from 2012 to 2022. The absolute number of women per group captures the extension of, for example, HPV‐based screening over time as well as potential changes in risk factors as exemplified by test‐positive results. We then calculated the proportion of cervical cancer cases diagnosed according to screening history group and the age‐standardized incidence rate per 100,000 person‐years by group stratified by calendar year. The age‐standardized incidence rate per 100,000 person‐years was calculated as the number of cervical cancer cases in a particular screening history group in a specific year divided by sum of the person‐years of all women in this screening history group in the year (standardized to the age distribution in Sweden for the year 2012). Each woman's contributing time of a calendar year is defined as entering the year from 1st of January, until diagnosis of invasive cervical cancer, death, emigration or 31st of December of the year, whichever came first. The trend of the incidence rates over calendar years could reflect the change of the performance of laboratories or the change of the effectiveness of positive finding management. Ninety‐five percent confidence intervals to accompany the age standardized incidence rates stratified by screening history group were calculated with the methods according to Breslow and Day.^15^

## 3 RESULTS

### 3.1 Cervical cancer cases 2018–2022 and their potential process‐related history

There were between 499 and 551 cervical cancer cases diagnosed per year in the years 2018–2022 for which medical chart re‐review was possible. In total, 2664 cervical cancer cases were included after the review. Twenty‐seven‐point 3% of cases were diagnosed at a microinvasive stage (i.e. stage IA), 32.6% of cases were diagnosed at localized stage (IB), and 40.1% of cases were at advanced stage (II+). The proportion of SCC, AC, ASC, and rare types were 65.7%, 28.8%, 2.4%, and 3.1%, respectively. Among the cases, 50.2% had a history of screening non‐participation, 26.2% had a history of potential laboratory performance issues (negative cytology in the preceding two intervals either as a primary test or as a triage after HPV positivity), and the remaining 23.5% had a history of potential clinical management issues. The distribution of screening‐chain performance issues shows a changing pattern when the primary screening test is shifted from cytology to HPV‐based screening. There has been a shift from false negative cytology as primary screening analyses to false negative cytology in triage of HPV‐positive screening results (Table 2).

**TABLE 2:** Characteristics of cervical cancer cases 2018–2022 after review.

	2018	2019	2020	2021	2022	Total
Number of cervical cancer cases after review	551	541	549	524	499	2664
Thereof number of cases in the cohort analysis in women ages 29–84	485	473	492	473	462	2385
FIGO stage (*N*, %)						
IA	149 (27.0)	131 (24.2)	145 (26.4)	157 (30.0)	146 (29.3)	728 (27.3)
IB	215 (39.0)	219 (40.5)	155 (28.3)	149 (28.4)	130 (26.1)	868 (32.6)
II+	187 (33.9)	191 (35.3)	249 (45.4)	218 (41.6)	223 (44.7)	1068 (40.1)
Histopathological type (*N*, %)						
Squamous cell carcinoma	353 (64.1)	361 (66.7)	360 (65.6)	343 (65.5)	332 (66.5)	1749 (65.7)
Adenocarcinoma	161 (29.2)	143 (26.4)	160 (29.1)	163 (31.1)	141 (28.3)	768 (28.8)
Adenosquamous carcinoma	15 (2.7)	17 (3.1)	9 (1.6)	7 (1.3)	16 (3.2)	64 (2.4)
Rare type carcinoma	22 (4.0)	20 (3.7)	20 (3.6)	11 (2.1)	10 (2.0)	83 (3.1)
Potential process‐related issues (*N*, %)						
Screening non‐attendance	277 (50.3)	253 (46.8)	283 (51.5)	285 (54.4)	238 (47.7)	1336 (50.2)
Lab performance	126 (22.9)	139 (25.7)	141 (25.7)	144 (27.5)	149 (29.9)	699 (26.2)
Management of abnormal findings	148 (26.9)	147 (27.2)	125 (22.8)	95 (18.1)	112 (22.4)	627 (23.5)
Mode of detection (*N*, %)						
Screening	259 (47.0)	245 (45.3)	267 (48.6)	235 (44.8)	258 (51.7)	1264 (47.4)
Symptoms	292 (53.0)	296 (54.7)	282 (51.4)	289 (55.2)	241 (48.3)	1400 (52.6)

### 3.2 Cervical cancer incidence in the population 2012–2022 by screening history

For cases diagnosed between 2012 and 2022, registry‐based data were available to evaluate incidence by screening history. Women ages 29–84 years were included since women younger than 29 did not have two full screening intervals to analyze. During 2012–2022, the proportion of women who were unscreened in the past two screening intervals decreased from 17.7% to 14.2%, corresponding to about 20% decrease. Women who were irregularly screened decreased from 35.3% to 23.9%, that is, 32% decrease. The proportion of women who were screened with only cytology normal in two intervals decreased from 42.7% to 16.7%, corresponding to 60% decrease. In contrast, women who were tested with HPV, either negative or positive, has largely increased. This reflects the switching from primary cytology to primary HPV screening. Meanwhile, women who had ever had any low‐ or high‐grade cytological abnormalities in last two intervals also increased from 4.0% to 6.6% corresponding to 65% increase. The proportion of screening detected abnormalities (i.e. women who had any cytology abnormal result(s) in past two intervals, including abnormalities from cytology‐primary screening and the cytology triage in HPV‐based screening) that were not managed according to guidelines increased from 4.0% to 6.6%, possibly reflecting challenges in prioritization and capacity (Table 3).

**TABLE 3:** Number of women ages 29–84 diagnosed with invasive cervical cancer in Sweden by screening history and calendar year in past two screening intervals.

Screening history classification	Number of women with invasive cervical cancer/Total number of women in the screening history category, *n*/*N*										
	(Proportion of all the cancer cases; proportion of the population in the screening history category, %), by calendar year										
	2012	2013	2014	2015	2016	2017	2018	2019	2020	2021	2022
Unscreened	135/525,593 (33.0; 17.7)	140/518,248 (34.3; 17.4)	141/512,742 (30.8; 17.1)	154/514,175 (30.4; 16.9)	139/506,704 (29.6; 16.5)	166/503,980 (35.2; 16.3)	159/496,995 (32.8; 15.8)	143/490,816 (30.2; 15.4)	157/481,221 (31.9; 14.9)	141/471,817 (29.8; 14.5)	143/466,810 (31.0; 14.2)
Irregularly screened	160/1,045,031 (39.1; 35.3)	129/1,033,891 (31.6; 34.7)	139/1,039,474 (30.2; 34.6)	157/1,060,940 (31.0; 35.0)	159/1,048,006 (33.9; 34.2)	142/1,022,919 (30.1; 33.0)	145/1,007,335 (29.9; 32.1)	144/978,037 (30.4; 30.8)	144/920,055 (29.3; 28.6)	109/885,424 (23.0; 27.2)	123/784,717 (26.6; 23.9)
Cytology normal	68/1,265,942 (16.6; 42.7)	89/1,296,735 (21.8; 43.5)	119/1,303,251 (26.0; 43.4)	114/1,283,793 (22.5; 42.3)	103/1,252,419 (22.0; 40.9)	101/1,242,972 (21.4; 40.1)	103/1,182,984 (21.2; 37.7)	104/1,069,212 (22,0; 33.6)	88/916,173 (17.9; 28.4)	87/727,634 (18.4; 22.4)	55/547,543 (11.9; 16.7)
HPV negative in both intervals	0/46 (0.0; 0.0)	0/40 (0.0; 0.0)	0/327 (0.0; 0.0)	0/782 (0.0; 0.0)	0/1074 (0.0; 0.0)	0/1597 (0.0; 0.1)	0/3402 (0.0; 0.1)	0/18,875 (0.0; 0.6)	0/44,232 (0.0; 1.4)	0/89,730 (0.0; 2.8)	5/149,013 (1.1; 4.5)
HPV negative in one interval	0/5990 (0.0; 0.2)	1/8313 (0.2; 0.3)	0/16,243 (0.0; 0.5)	1/31,293 (0.5; 1.0)	2/95,010 (0.4; 3.1)	3/154,389 (0.6; 5.0)	6/256,420 (1.2; 8.2)	5/409,853 (1.1; 12.9)	8/618,373 (1.6; 19.2)	19/814,131 (4.0; 25.0)	22/1,046,795 (4.8; 31.9)
HPV positive and cytology negative	0/844 (0.0; 0.0)	0/1195 (0.0; 0.0)	1/1828 (0.2; 0.1)	0/3030 (0.0; 0.1)	4/7259 (0.9; 0.2)	7/11,401 (1.5; 0.4)	11/18,151 (2.3; 0.6)	12/27,748 (2.5; 0.9)	27/40,434 (5.5; 1.3)	53/52,779 (11.2; 1.6)	48/64,355 (10.4; 2.0)
Screen‐detected abnormality^a^	46/119,718 (11.2; 4.0)	49/124,978 (12.0; 4.2)	58/132,127 (12.7; 4.4)	79/139,760 (15.6; 4.6)	61/151,735 (13.0; 5.0)	53/162,844 (11.2; 5.3)	61/173,238 (12.6; 5.5)	63/184,711 (13.3; 5.8)	67/199,560 (13.6; 6.2)	60/209,454 (12.7; 6.4)	59/217,887 (12.8; 6.6)
Screen‐detected HPV positivity without follow‐up	0/68 (0.0; 0.0)	0/66 (0.0; 0.0)	0/105 (0.0; 0.0)	1/217 (0.0; 0.0)	1/349 (0.2; 0.0)	0/593 (0.0; 0.0)	0/609 (0.0; 0.0)	2/819 (0.4; 0.0)	1/914 (0.2; 0.0)	4/1439 (0.8; 0.0)	7/6328 (1.5; 0.2)
Total target population by year	2,963,232	2,983,466	3,006,097	3,033,990	3,062,556	3,100,695	3,139,134	3,180,071	3,220,962	3,252,408	3,283,448

^a^ Cytology abnormal result(s) in past two intervals, including abnormalities from cytology‐primary screening and the cytology triage in HPV‐based screening.

Incidence rates (IR) of cervical cancer by different screening history groups exhibited different patterns from 2012 to 2022. The IR in women who were screened with only cytology normal results in both two intervals increased from 5.5 to 12 per 100,000 person‐years, with a statistically significant increasing trend (*p*‐value, *p* < .0001). The IRs in women who had HPV negative test result(s) were mostly lower than 4 per 100,000 person‐years, whereas IRs in women who had HPV positive with only normal cytology results or lacking further testing were as high as 40–80 and 100–250 per 100,000 person‐years, respectively. The IRs were much higher than those in women who have had any cytology abnormalities in the past two screening rounds, which were mostly between 30 and 40 per 100,000 person‐years and had a statistically significant decreasing trend from 2012 to 2022 (*p* = .0049) (Table 4).

**TABLE 4:** Age‐standardized incidence rate of cervical cancer among women ages 29–84 according to screening history in the past two screening intervals, by calendar year.

Screening history classification	Calendar year (incidence rate per 100,000 person‐years, 95% confidence interval)										
	2012	2013	2014	2015	2016	2017	2018	2019	2020	2021	2022
Unscreened	27.4 (22.5–32.3)	29.5 (24.5–34.6)	30.0 (24.9–35.2)	32.8 (27.5–38.1)	29.7 (24.6–34.7)	36.3 (30.7–41.9)	34.0 (28.6–39.4)	31.1 (25.9–36.3)	35.3 (29.7–41.0)	30.7 (25.5–35.9)	33.9 (28.2–39.5)
Irregularly screened	15.6 (13.2–18.1)	12.8 (10.6–15.0)	13.6 (11.4–15.9)	15.5 (13.0–17.9)	15.9 (13.4–18.4)	14.2 (11.8–16.6)	14.8 (12.3–17.3)	15.3 (12.7–17.9)	16.5 (13.6–19.4)	13.6 (10.9–16.4)	18.1 (14.6–21.7)
Cytology normal	5.5 (4.1–6.8)	7.2 (5.6–8.7)	9.2 (7.5–10.9)	8.8 (7.1–10.4)	8.5 (6.8–10.1)	8.4 (6.7–10.0)	8.7 (7.0–10.4)	9.6 (7.8–11.5)	9.7 (7.7–11.8)	12.6 (9.9–15.3)	11.5 (8.1–14.9)
HPV negative in both intervals							0.0 (0.0–0.0)	0.0 (0.0–0.0)	0.0 (0.0–0.0)	0.0 (0.0–0.0)	2.8 (0.2–5.4)
HPV negative in one interval							2.0 (0.4–3.5)	1.0 (0.1–1.9)	1.1 (0.3–1.9)	4.3 (0.0–9.0)	2.9 (0.4–5.5)
HPV positive and cytology negative							48.4 (18.6–78.2)	40.0 (16.3–63.8)	58.7 (35.0–82.4)	81.7 (58.5–104.9)	56.7 (39.9–73.6)
Screen‐detected abnormality	37.0 (25.5–48.5)	38.5 (27.0–50.0)	46.6 (33.7–59.5)	60.4 (45.9–74.9)	42.5 (30.9–54.1)	31.1 (22.0–40.3)	39.2 (28.2–50.3)	38.2 (27.6–48.7)	36.6 (26.6–46.6)	34.3 (24.4–44.2)	30.1 (21.4–38.8)
Screen‐detected HPV positivity without follow‐up							0.0 (0.0–0.0)	226.8 (0.0–543.7)	105.8 (0.0–313.1)	258.6 (2.5–514.8)	101.7 (22.0–181.4)

## 4 DISCUSSION

### 4.1 Main findings and interpretations

This framework for a nation‐wide register‐based cervical cancer audit includes all annual cases by age, FIGO stage, and histological type. Medical chart re‐review was possible for cases between 2018 and 2022 while registry‐based data were available for the whole time period (2012–2022). It demonstrates the absolute burden of cervical cancer and the incidence rate by screening history, in the transition from cytology to HPV‐based screening. The results reflect differing risks as screening methods change in a real‐life program and demonstrate aspects of the screening chain that need optimization. In this study we have analyzed screening status and history in the preceding two intervals for cervical cancer cases between 2012 and 2022. We have estimated the incidence rate of cancer by screening history classification. This model can be used annually in a replicable way and thereby provide stratified feedback to those who are responsible for the guidelines and the implementation.

HPV‐based screening was recommended in 2015, but the programmatic transition did not start until 2017 and it was not fully implemented until 2021. During the study period 2012–2022, there was a decreasing number of women screened by cytology with normal results and an increased number of women screened by HPV test with negative test results which reflects the gradual switching from cytology‐ to HPV‐based screening in recent years. Screening attendance has been high in Sweden for many years but the main risk factor for cervical cancer is still non‐attendance (Table 2).^6^, ^7^, ^12^ The level of non‐attendance has been relatively stable over time with gradual increases in attendance year on year,^9^ most significantly when switching from clinician‐based HPV sampling to self‐sampling during the pandemic.^16^ The switch to HPV‐based screening has been examined in a randomized health policy trial that demonstrated a slightly higher attendance rate among women invited to HPV screening as compared to cytology.^17^

The absolute number of cancer cases after a negative cytology are declining as primary cytology is no longer recommended as a screening test; however, the increased incidence rate of cervical cancer among women who had been screened by cytology only with normal results in the past two intervals strongly suggests that the quality of the cytology testing has decreased.^18^ This declining protection of normal cytology has been investigated in previous studies and found to correlate with an increasing proportion of false negative cytologies, as revealed by a systematic re‐review of normal cytologies taken before invasive cancer.^19^ Cytology was initially retained as a triage strategy for HPV positives to increase specificity, but we nuanced the policy in 2025 and now women with HPV 16/18 positivity in screening are referred to colposcopy regardless of cytology results as the risk in this group was substantial even when cytology negative.^20^

Cancers after a negative HPV result are very rare despite the large and increasing proportion of the population tested. The incidence rates in women who were tested HPV negative were at or lower than the WHO elimination threshold of 4 per 100,000 person‐years. Attendance and cytology tests in previous intervals are of little importance if a woman has attended in the last interval with a negative HPV. However, the high incidence rate in women who had positive HPV and negative cytology triage indicates that the quality decrease of cytology affects this aspect of the HPV‐based screening as well.^20^ In 2021 and 2022, 10% of cases were found in this group which highlights that moving away from cytology as a triage method should also be considered.

Prevention through detection of HPV and early diagnosis of a precursor or cancer is critical for achieving reductions in incidence and mortality. Our findings among the clinical records review suggest that half of the cancer cases diagnosed from 2018 to 2022 were among women who did not attend routine screening, approximately one‐fourth were among women with a history of laboratory performance issues, and another one‐fourth of cases had a history of issues relating to the clinical management of abnormal findings detected through screening. HPV screening is highly sensitive, and stratification of results is necessary to achieve prioritization of resources for timely follow‐up and balance benefits and harms of following infections with varying risk.^21^, ^22^

### 4.2 Strengths and limitations

The strength of this annual audit framework of cervical cancer cases is the use of individual level linkage of the longitudinal population registry with confirmation and validation by review of medical charts, screening registry and cancer registry with almost 100% of coverage in the entire population, which enables a credible estimation of the attributions of cervical cancer to different issues in the screening chain with year‐on‐year comparison. Nevertheless, it is worthwhile emphasizing the limitation that these measurements can only suggest potential deficiencies in the screening chain. To confirm the problems and devise programmatic solutions, further in‐depth investigations can be needed with a focus on details obtained through medical chart reviews. Given that this analysis focuses on incidence rates according to screening status and history, cases under the age of 30 years old are not addressed specifically. Anecdotally, cervical cancer cases under age 30 are still detected but rarely and are usually associated with HPV 16, 18, or 45 (high oncogenic types that are now vaccinated against in Sweden). As birth cohorts vaccinated in the school‐based program with high coverage age into screening, further reductions in infections and associated cervical cancer incidence are expected.^23^

## 5 CONCLUSIONS

Robust data, reproducible and comparable definitions and classifications of cervical cancer cases and screening history are needed to perform audits routinely. In this audit framework, we have used two quality registers with routinely collected data representing almost 100% coverage combined confirmation and validation through review of medical charts. Many countries have detailed cancer registers including FIGO‐stage, age of diagnosis of cancer and treatment as well as registers containing laboratory results about invitations, screening results, and histopathology allowing for translation or adaption of this framework into other settings. Once populations have been screened with HPV and vaccinated cohorts are more common in screening target ages, the risk for cervical cancer will decrease significantly. We also showed that the risk for cancer is related to screening history where there is a high risk for cancer when unscreened or poorly screened within the two previous screening intervals before cancer was diagnosed. Furthermore, there we also showed that there is an elevated risk when screened with cytology only and when the HPV‐positive screening test has a normal cytology at triage. This audit framework will therefore need continuous updates, mainly to refine the screening history groups to accommodate changes in potential risk factors and to find remaining pockets of risk in the target population. The audit procedure used gives valuable and comparable information of deficiencies in the screening program and can be used in the transition from cytology to HPV screening to understand changing risk in the population and provide feedback loops to the program.

## AUTHOR CONTRIBUTIONS

**Cecilia Kärrberg:** Conceptualization; methodology; writing – original draft; writing – review and editing; formal analysis; investigation; validation; project administration. **Penelope Gray:** Conceptualization; writing – review and editing; writing – original draft; formal analysis; methodology; investigation; validation. **Kristina Elfgren:** Conceptualization; methodology; investigation; writing – review and editing. **Hanna Milerad:** Conceptualization; methodology; investigation; writing – review and editing. **Bengt Andrae:** Conceptualization; methodology; data curation; investigation; validation; formal analysis; visualization; supervision; writing – review and editing. **Jiayao Lei:** Conceptualization; methodology; writing – review and editing; formal analysis. **Pär Sparén:** Conceptualization; methodology; software; supervision; writing – review and editing; data curation. **Joakim Dillner:** Conceptualization; methodology; supervision; funding acquisition; resources; writing – review and editing. **Jiangrong Wang:** Conceptualization; methodology; formal analysis; validation; investigation; supervision; project administration; writing – review and editing; writing – original draft; visualization. **K. Miriam Elfström:** Conceptualization; methodology; investigation; formal analysis; supervision; writing – review and editing; writing – original draft.

## FUNDING INFORMATION

NKCx is supported by grants (to JD) from the Swedish Association of Local Authorities and Regions as well as from the National Board of Health and Welfare.

## CONFLICT OF INTEREST STATEMENT

CK is PI for one study site in a multicenter international clinical trial of HPV vaccination, the trial sponsor, Imperial College London, received financial support from MSD, and has received reimbursement for sponsored lectures by Gedeon Richter. JW received part of their salary from a research grant from Merck Sharp & Dohme Corp. to the affiliated institution for other studies on HPV vaccination in Sweden. The other authors have no conflicts of interest to declare.

## ETHICS STATEMENT

This study was approved by an ethical review board from the Swedish Ethical Review Agency in 2011 (decision number: 2011/1026‐31/4).

## Data Availability

Aggregated data is presented in the tables. Individual‐level data can be shared by joakim.dillner@ki.se (https://nkcx.se/research_e.htm) upon reasonable request with ethical permission. Further information is available from the corresponding author upon request.
